# First record of the Miocene hominoid *Sivapithecus* from Kutch, Gujarat state, western India

**DOI:** 10.1371/journal.pone.0206314

**Published:** 2018-11-14

**Authors:** Ansuya Bhandari, Richard F. Kay, Blythe A. Williams, Brahma Nand Tiwari, Sunil Bajpai, Tobin Hieronymus

**Affiliations:** 1 Birbal Sahni Institute of Palaeosciences, Lucknow, India; 2 Department of Evolutionary Anthropology, Duke University, Durham, NC, United States of America; 3 Division of Earth and Ocean Sciences, Nicholas School of the Environment, Duke University, Durham, NC, United States of America; 4 Wadia Institute of Himalayan Geology, Dehra Dun, India; 5 Department of Earth Sciences, Indian Institute of Technology, Roorkee, Uttarakhand, India; 6 Northeastern Ohio Medical University, Rootstown, Ohio, United States of America; Ecole Normale Supérieure de Lyon, FRANCE

## Abstract

Hominoid remains from Miocene deposits in India and Pakistan have played a pivotal role in understanding the evolution of great apes and humans since they were first described in the 19^th^ Century. We describe here a hominoid maxillary fragment preserving the canine and cheek teeth collected in 2011 from the Kutch (= Kachchh) basin in the Kutch district, Gujarat state, western India. A basal Late Miocene age is proposed based on the associated faunal assemblage that includes *Hipparion* and other age-diagnostic mammalian taxa. Miocene Hominoidea are known previously from several areas of the Siwalik Group in the outer western Himalayas of India, Pakistan, and Nepal. This is the first record of a hominoid from the Neogene of the Kutch Basin and represents a significant southern range extension of Miocene hominoids in the Indian peninsula. The specimen is assigned to the Genus *Sivapithecus*, species unspecified.

## Introduction

**Fig 1:**
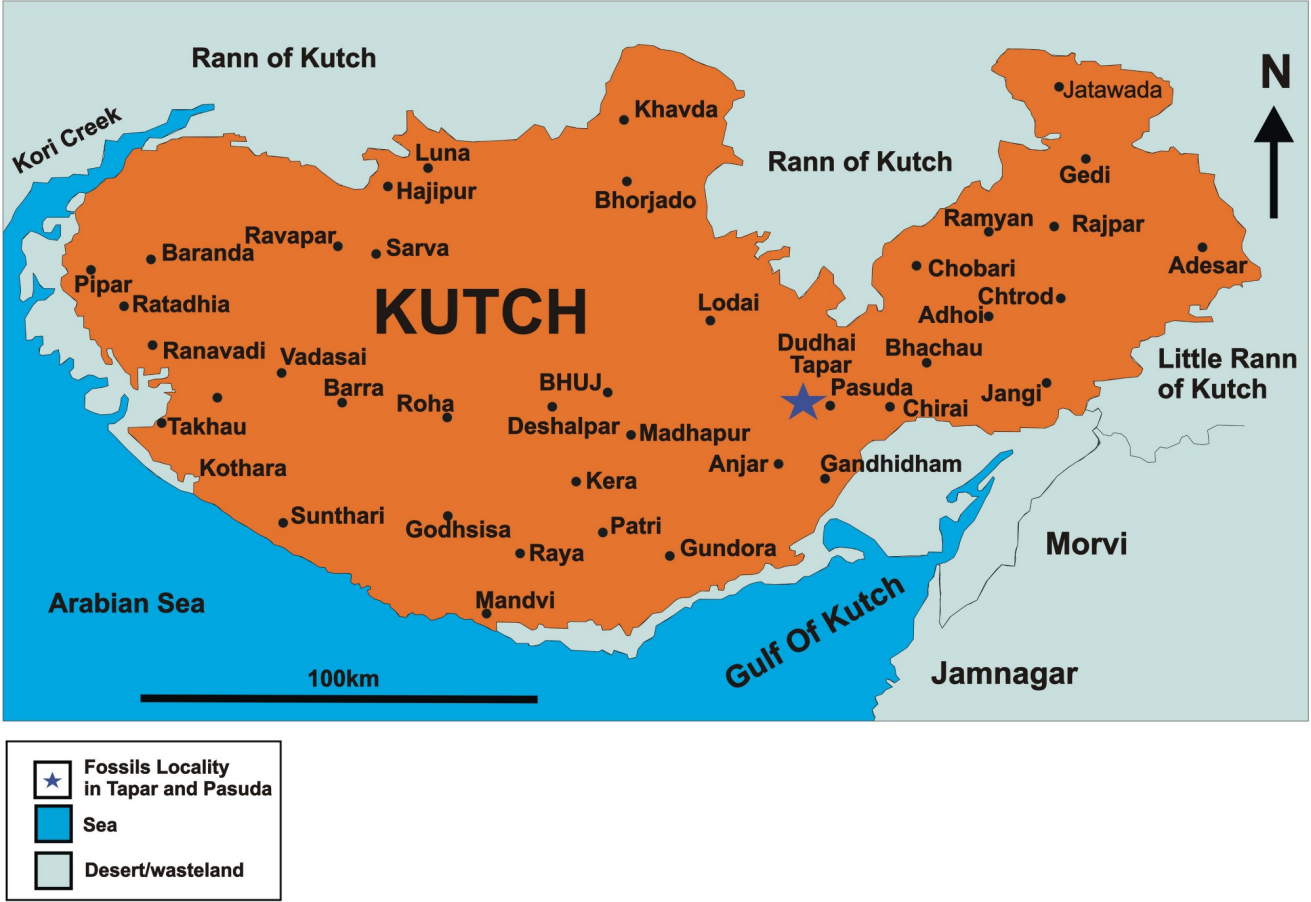
Map of the Kutch (= Kachchh) Peninsula surrounded by the Rann of Kutch and the Arabian Sea.

**Fig 2:**
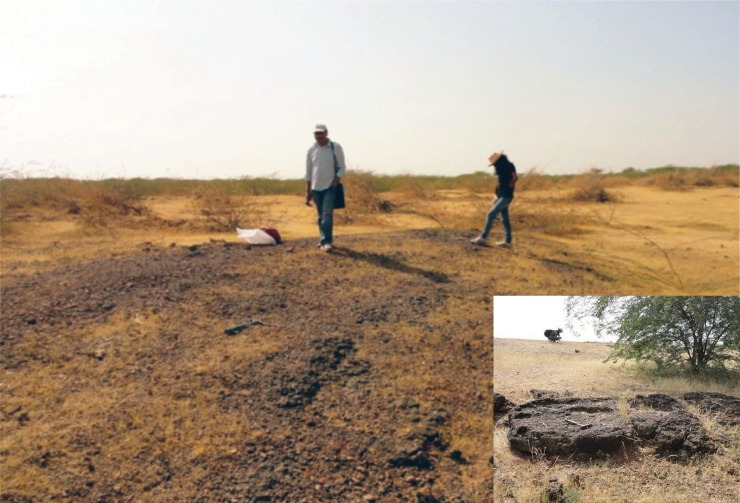
Field photographs of fossil-bearing exposures at Tapar, Gujarat state, western India. Inset, example of conglomerate exposure.

**Fig 3:**
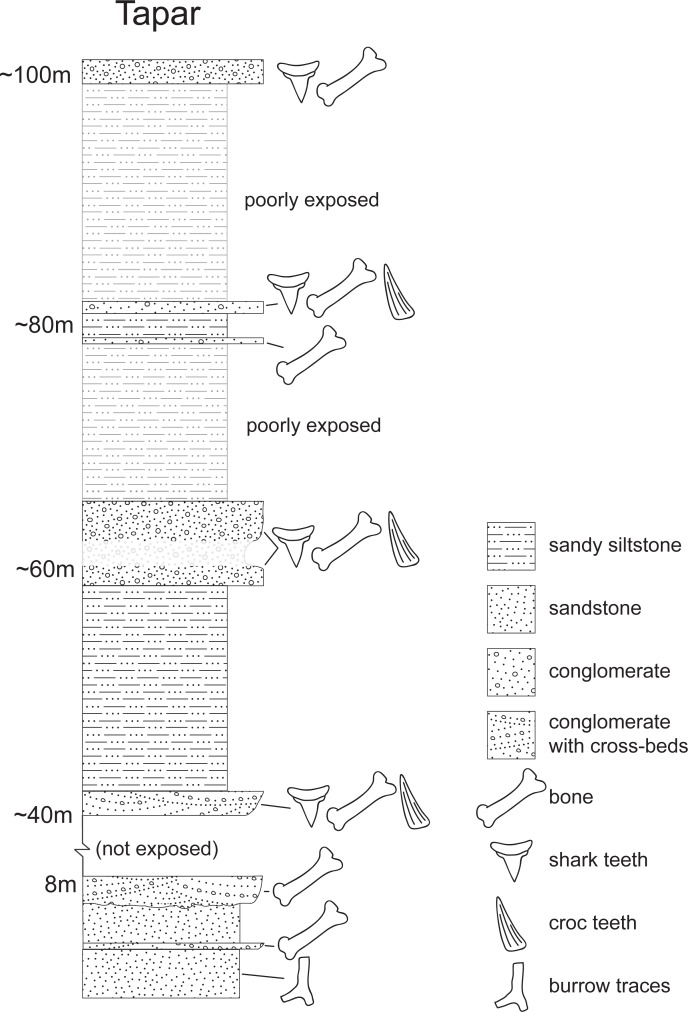
Miocene sediments exposed at locality Tapar III, Gujarat (~ 23° 15' 16" N, 70° 08' 50" E). The hominoid fossil came from the lower conglomerate bed at the 8-meter mark.

**Fig 4:**
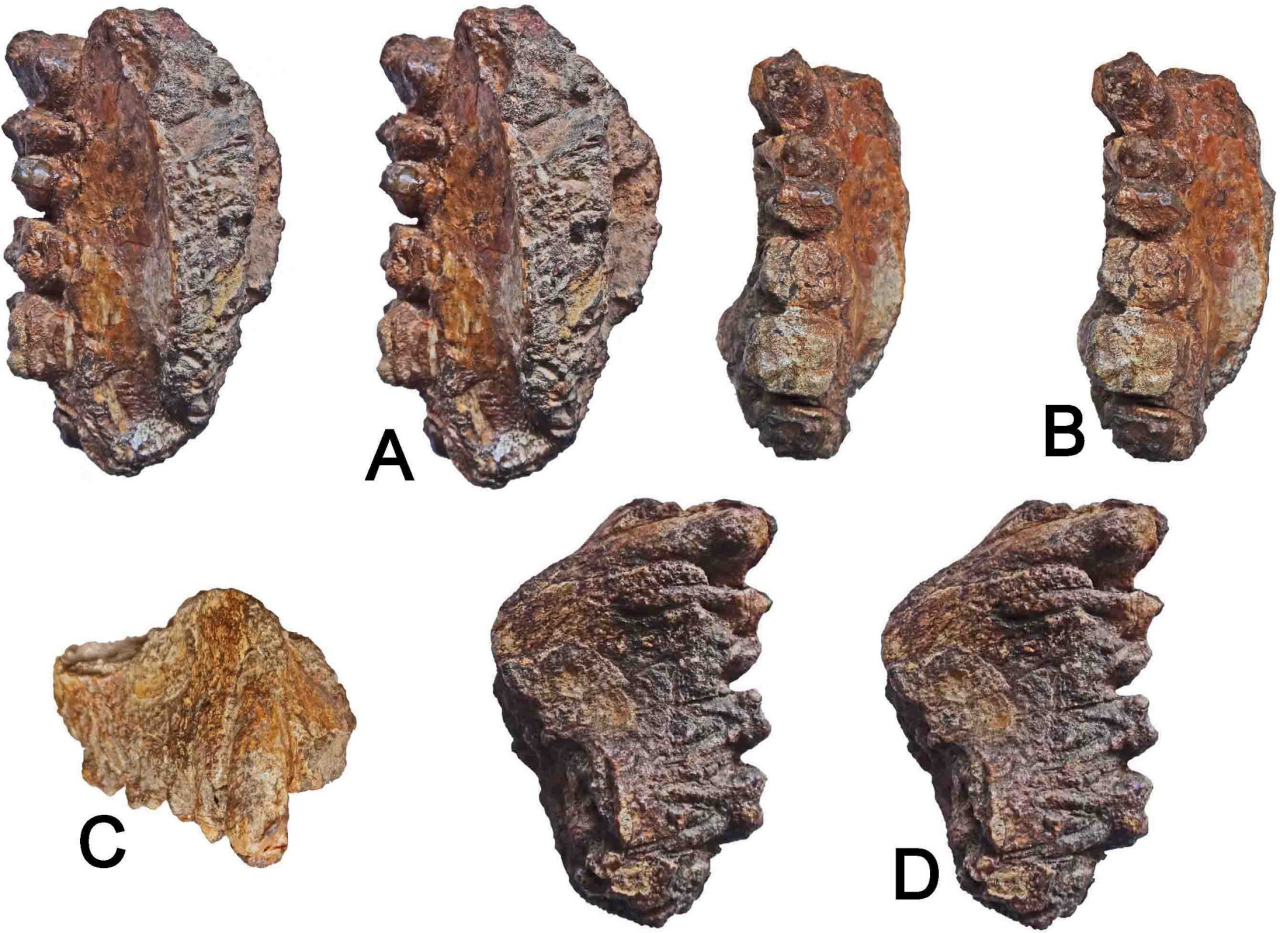
WIHG WIF/A 1099, right maxilla preserving Canine-M2. A. Stereopair of the palate and teeth viewed from lingual perspective. B. Stereopair of the palate and teeth viewed in occlusal perspective. C. The teeth and maxilla viewed frontally. D. Stereopair palate and teeth viewed from lateral (buccal) perspective. Image magnification is variable. Length of toothrow (C-M2) = 450 mm; specimen length = 575 mm.

Recent fieldwork in the Kutch (= Kachchh) district in the state of Gujarat, western India has expanded our knowledge of the Miocene mammalian faunas of the region [1, 2]. Vertebrate fossil remains recovered from the Pasuda and Tapar localities in the Bachau Taluka area of central Kutch Figs 1–3) during expeditions in 2011 and 2012 were described by Bhandari et al. [2]. Amongst the collections reported is a hominoid maxillary fragment that is the subject of this report. The new specimen (WIHG WIF/A 1099, Fig 4) is from the Tapar locality, younger than 10.8 Ma based on the presence of *Hipparion* (*sensu lato*) [3]. The hominoid specimen is a maxilla with C-M2, with roots of M3 and likely belongs to *Sivapithecus* Pilgrim, 1910. *Sivapithecus* and other Miocene hominoid taxa are best known from the Siwalik group of India and Pakistan approximately 10 degrees of latitude and more than 1000 kilometers to the north of Kutch. Prior to these collecting efforts of A. Bhandari and colleagues, no hominoid specimens had been recovered from so far south on the subcontinent.

Hominoid remains from Miocene deposits in the Siwaliks of India and Pakistan have played a pivotal role in understanding the evolution of great apes and humans since the description of “*Paleopithecus*” = *Sivapithecus sivalensis* from the Potwar Plateau of Pakistan [4]. Many hominoid taxa (including “*Ramapithecus”*, a taxon that was considered a possible human ancestor but is now thought to represent small specimens of *Sivapithecus* [5]), were named by researchers collecting in the Siwaliks. Today, four Miocene hominoid taxa commonly are recognized in two large-bodied sympatric genera; the larger of which is *Indopithecus* (or *Gigantopithecus*, see below) and the smaller *Sivapithecus*. *Indopithecus/Gigantopithecus* is reported from Hari Talyangar, Himachal Pradesh State, India [6]. Pilgrim [7] named the taxon *Sivapithecus giganteus* based on an isolated second or third molar from the Nagri Formation. Later, Simons and Chopra [8] described *Gigantopithecus bilaspurensis* based on a mandible from near Hari Talyangar. These two larger specimens probably represent the same species, *Gigantopithecus giganteus* [9], although others call the Siwalik taxon *Indopithecus giganteus* in recognition of its distinctiveness from Pleistocene *Gigantopithecus* of China and Viet Nam [6, 10–12], a convention we will follow in this paper. Three other commonly recognized Siwalik taxa are species of *Sivapithecus*: *S*. *indicus*, *S*. *parvada*, *and S*. *sivalensis*. A fourth species known from very limited material, *S*. *simonsi*, possibly equivalent to *S*. *hysudricus*, is recognized by some authors [13–15].

The oldest hominoid remains in Indo-Pakistan are dated at about 12.7 Ma and the youngest at about 8.6 Ma (see below). In the past, both *Sivapithecus* and *Indopithecus* were argued to have been on or near the human lineage; see historical reviews in [9, 16]. Some researchers posit a sister-taxon relationship between *Sivapithecus* and *Pongo* based on shared cranial and dental features [9, 17]. *Sivapithecus* lacks the suspensory features of the limbs found in extant great apes of Africa and Asia [18, 19]. Therefore, a *Pongo*-*Sivapithecus* sister-group hypothesis would require that anatomical features associated with suspensory behavior in extant African and Asian great apes evolved independently, a view that some find implausible [20, 21]. Chinese *Lufengpithecus* also is thought by some specialists to be closely related to *Sivapithecus* and *Pongo* [10] but others disagree [22], as is *Khoratpithecus* from Thailand [23], and *Ankarapithecus* from Turkey [9, 10, 17].

Fossil hominoids appear in the Chinji Formation, Potwar Plateau of Pakistan (Siwalik Series), as early as 12.7 Ma [24]. In Pakistan, *Sivapithecus* continues to occur intermittently in the Siwalik series (Nagri and Dhok Pathan Formations) up to as young as 8.5 Ma [25]. In India, the bulk of *Sivapithecus* (*sensu lato*) comes from the area around Hari Talyangar and from the Ramnagar Basin of Jammu and Kashmir [9]. The Hari Talyangar material ranges in age from about 9.2 to 8.6 Ma [6]. Based on faunal correlation, the Ramnagar material is older, penecontemporaneous with that from Chinji [26].

## Geology and fauna

Deposition in the dominantly marine Kutch Basin on the northwestern margin of Gujarat state (Fig 1) was initiated in the Jurassic, when India separated from Madagascar. Subsequently, it was subjected to triple-junction rifting, which also affected the Cambay and Narmada rift zones [27, 28]. Reactivation of the western margin rifts has occurred episodically, leading to crisscrossing faults in the Kutch area. The Deccan continental flood basalts dome the central part of Kutch. Circumferentially outwards from these centrally located volcanic flows lie a well exposed crescentic Tertiary sequence spanning most of the Cenozoic. In southwestern Kutch, the Paleogene is marked by lagoonal and near-shore marine sedimentation with Middle Eocene shales and limestones containing age-diagnostic foraminifera and nannofossils, overlying laterally interfingering with lignite deposits of Early Eocene age. Mainly near-shore marine and lagoonal deposition continued throughout the Neogene with the exception of short regressive phases, primarily during the Middle and Late Miocene. These Neogene deposits produce terrestrial vertebrate fossils, including mammals [2, 29].

The depositional context of the mammalian fossils of Kutch, including those of Tapar and nearby Pasuda, is difficult to interpret due to their recovery from calcareous nodules in conglomerates found in close association with marine mollusks, fish, crocodiles, and turtles [2]. These conglomerates may represent transgressive lags that have exhumed and concentrated siderite-cemented nodules (with mammal fossils) from earlier parent rock onto a flooding surface during a rise in sea level, thus complicating the biostratigraphic interpretation. The Tapar hominoid specimen was found in such a calcareous/ferruginous conglomerate.

The Tapar locality was initially thought by Bhandari et al. [1] to date to about 16 Ma on the basis of inferred correlation with the Khari Nadi Formation. The correlation was problematic, as the type area of the Khari Nadi Formation is in western Kutch whereas the Tapar (and nearby Pasuda) beds are in central Kutch, and the central and western deposits are discontinuous. Recovery of *Hipparion* (*sensu lato*) and other mammalian fossils led Bhandari et al. [2] to propose a revised date for the Tapar and Pasuda beds of ~11–10 Ma (basal Late Miocene). Dating of the first appearance of *Hipparion* equid fossils in the Old World may be complicated by multiple events that occurred in different places [30–33]. The *Hipparion* first Asian occurrence is best established in the Potwar Plateau where it occurs in the normal-polarity interval Chron C5n dated to ~ 10.7 Ma [25]. Thus, the presence of *Hipparion* (*sensu lato*) at Tapar indicates a datum range of 11–10 Ma or younger. The Tapar record of the artiodactyl *Dorcatherium minus* is consistent with an age estimate of 11–10 Ma, as these taxa are also typical of the Chinji-Nagri deposits of the Potwar Plateau, Pakistan [2]. The presence of *Sanitherium schlagintweiti* provides an upper bound for time-averaging at Tapar, as the last occurrence of this taxon in Chinji deposits is placed at ~14 Ma [34].

## Materials and methods

No field permits were required to perform this research. WIHG WIF/A 1099 (Wadia Institute of Himalayan Geology, Dehra Dun, India)was recovered during collecting efforts by A. Bhandari and colleagues in 2011. Collections made during the 2011 and 2012 field seasons were described by Bhandari et al. [2], with a brief mention of specimen WIHG WIF/A 1099. Measurements of the specimen were made with dial calipers to the nearest one-tenth mm. The specimen was CT scanned at the University of Texas High- Resolution X-Ray Computed Tomography Facility. The jpeg stacks and details of the scanning protocol are linked to the doi of this paper and posted and available for download at www.MorphoSource.org. For comparative purposes, in a Supporting information (S1 Table), we provide the dimensions of the cheek teeth of a sample of extant great ape species *Pan* and *Pongo* (data from Plavcan [35] and of all Indo-Pakistan *Sivapithecus* specimens available to us. Measurements of most of these specimens were made by RFK except as noted for a few specimens, which come from the literature. The individual in this manuscript has given written informed consent (as outlined in PLOS consent form) to publish these case details.

## Abbreviations

Upper teeth are indicated with capital letters; lower teeth with lower case letters, vis, M1 for the upper first molar and m1 for the lower first molar.

Museum designations are as follows (most of these designations occur only in S1 Table):

BMNH         Natural History Museum, London

BSPhG       Bayerische Staatssammlung für Paläontologie und historische Geologie

GSI           Geological Society of India, Calcutta

GSP           Geological Survey of Pakistan, Islamabad, Pakistan.

ONGC         Oil and Natural Gas Commission, Dehra Dun, India.

PUA           Dep. of Anthropology, Panjab University, Chandigarh, India

SFP           Saketi Fossil Park [13]

SM             Abteilung Palaoanthropologie, Naturforschungsinstitut Senckenberg, Germany

WIHG         Wadia Institute of Himalayan Geology, Dehra Dun, India

YPM           Yale Peabody Museum, New Haven, Connecticut, USA

## Systematic description

Order: Primates Linnaeus, 1758

Superfamily: Hominoidea Gray, 1825

Genus: *Sivapithecus* Pilgrim, 1910

Type species: *Sivapithecus sivalensis* Pilgrim, 1910

*Sivapithecus* sp.

### Anatomical comparisons

**Fig 5:**
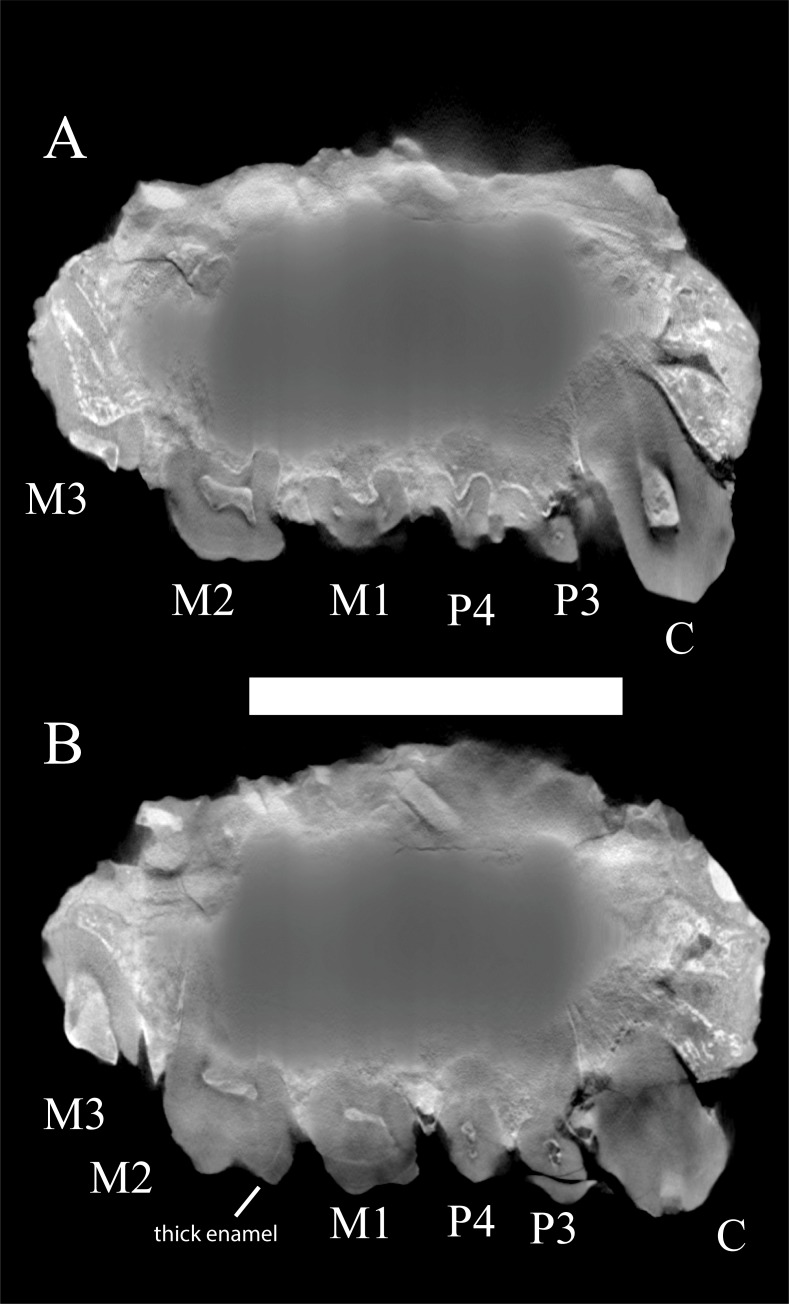
Two high resolution micro-CT parasagittal sections of WIHG WIF/A 1099, with teeth identified. A. The root structure of the canine. B. The enamel thickness on M1. Scale bar equals 15 cm.

WIHG WIF/A 1099 is the right maxilla of an adult individual including parts of the alveolar bone and the facial surface approaching the root of the zygomatic process and a lateral part of the palatal process, but not reaching the mid-sagittal plane (Fig 4). Canine to M2 crowns and M3 roots are preserved in the specimen but in a damaged condition, as detailed below. The external features of the specimen were examined. Additionally, high-resolution CT-imaging reveals some details of the tooth enamel and root structure (Fig 5) but mineralization of the fossil bone precludes determination of most details of the internal anatomy.

#### Maxilla

Due to postmortem breakage, the curvature of the tooth row, and the width, and depth of the palate, the size and shape of the incisive foramen or posterior palatine foramen, and the depth of the facial process cannot be established. Mineralization of the fossil bone precludes determination of the size of the maxillary sinus from CT images.

The CT sections reveal the apex of the root socket for the upper second incisor and suggest that there was a diastema between the lateral incisor and canine. A canine fossa appears to be present, but its size and extent is indeterminant.

#### Dentition

Dimensions of C-M2 of WIHG WIF/A 1099 are presented in Table 1. For comparative purposes the dimensions of all published (or available) maxillary teeth of *Sivapithecus* species and of samples of male and female specimens of extant *Pan troglodytes* and *Pongo pygmaeus* are given in S1 Table. The enamel caps are damaged but not so profoundly that we cannot make reasonable estimates of the dimensions of the teeth and judge overall enamel thickness in many places. All teeth from P4 to M2 are small compared to the available *Sivapithecus* sample, being most comparable to Hari Talyangar specimens YPM 13799 and GSI D 185 and are certainly at the smaller end of the range of the Hari Talyangar specimens.

**Table 1:** WIHG WIF/A 1099, right palate. Measurements are in mm or square mm. Owing to postmortem damage, measurements are estimated, based on a reconstructed shape, using the preserved parts of the enamel.

Tooth position	mesiodistal length	buccolingual breadth
Canine	11.9	—
P3	7.0	—
P4	6.4	10.0
M1	9.4	10.9
M2	9.9	12.4
P4 area	64	
M1 area	102	
M2 area	123	
Ratio, P4 area to M1 area	0.62	
Ratio, M1 area to M2 area	0.83	
M1 shape	0.87	

Canine: The canine has a robust crown and root (Figs 4 and 5). A mesial ridge is present, forming the mesiobuccal side of a shallow mesial groove that terminates cervically against the furthest mesial extent of the crown; the ridge and groove do not extend onto the root. A blunt crest (postparacrista) leads distally from the crown apex and would have occluded against the lower third premolar. The projective height of the canine is not determinate because of postmortem damage and wear to the individual during life. The ratio of the canine to M1 mesiodistal lengths is 1.26, which falls in the middle of the range for *Sivapithecus* specimens that preserve the two teeth (n = 9; range 1.04 to 1.47). Such a range is not unexpected for a dimorphic ape; in our sample, *Pongo* ranges from 0.99 to 1.66; *Pan* ranges from 0.97 to 1.55 (*Pongo* and *Pan* data in S1 Table).

Premolars: P3 and P4 of WIHG WIF/A 1099 have two buccal and one lingual root. The buccal part of P3 is broken and the tooth’s breadth cannot be reliably estimated. The two teeth are similar in preserved morphology, with low, rounded cusps and gently sloping occlusal surfaces. Mesial and distal enamel cingula cannot be identified due to functional wear and postmortem damage. P4 has a faint and blunt lingual cingulum; the lingual enamel is not preserved on P3. When compared with molar size, the premolars are similar in proportions to *Sivapithecus*. The ratio of P4 area to M1 area is 0.62 (Table 2). This value falls near the mean for known shape ranges for *Sivapithecus* (0.49 to 0.70).

**Table 2:** Ratios of premolar to molar dimensions for *Sivapithecus* spp., *Pongo pygmaeus*, and *Pan troglodytes*. Areas are computed as mesiodistal length times buccolingual breadth. Data for *Pongo* and *Pan* comes from Plavcan [35]. Data for *Sivapithecus* calculated from complete specimens enumerated in S1 Table.

Taxon	P4 area to M1 area				M1 area to M2 area			
	Sample size	Mean	Range	CV	Sample size	Mean	Range	CV
*Pongo pygmaeus*	33	0.78	0.59 to 0.88	7.96	33	0.97	0.81 to 1.18	9.36
*Pan tr*. *troglodytes*	35	0.64	0.54 to 0.87	9.37	35	1.00	0.86 to 1.14	6.27
*Sivapithecus* spp.	13	0.62	0.49 to 0.70	8.53	15	0.82	0.68 to 0.92	10.39
WIHG WIF/A 1099	1	0.62	—	—	1	0.84	—	—

Molars: The first and second molars are preserved, although each has lost considerable enamel. Only the roots of the third molar remain. Allowing for the loss of enamel and post-mortem crown abrasion, the following salient points can be made: As in *Sivapithecus* and most other Miocene apes, the molar crowns are bunodont with poorly developed cresting. As in *Sivapithecus*, the dentine horns do not penetrate far into the enamel cusps (Fig 5). By comparison, the dentin horns of *Dryopithecus fontani* extend far into the cusp tips [36]. As in *Sivapithecus*, there is no evidence for a lingual or buccal cingulum or cuspal cingular elements. This contrasts with the condition seen in late Early Miocene taxa, which have distinct cingula [10].

On M1-2 the four cusps are approximately equal in size, although the hypocone may be slightly smaller than the protocone. The M2 metacone is smaller than the paracone whereas the two cusps are comparable in size on M1. Each tooth is roughly quadrangular with the mesiodistal dimension 86% of the buccolingual. The mean for 33 specimens of *Sivapithecus* is 0.87.

As with other *Sivapithecus*, in WIHG WIF/A 1099, M2 is about 20% larger than M1.

Although the enamel of the molar crowns is eroded in most places, its full thickness is preserved in a few places, particularly on the buccal aspect of M2. Where it can be compared directly (Fig 5), the enamel is very thick, comparable to that that seen in Siwalik *Sivapithecus* [37, 38].

**Fig 6:**
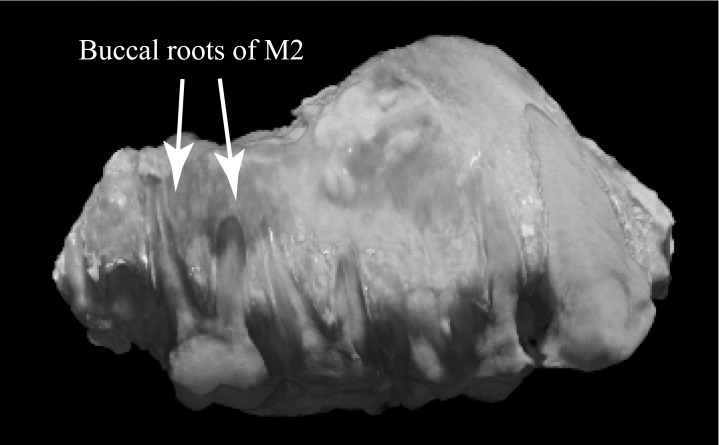
Buccal view of WIHG WIF/A 1099, composite CT image. Length of toothrow (C-M2) = 450 mm.

In lateral view, M1 and M2 have two buccal molar roots as in *Sivapithecus* and most hominids. The roots appear long (relative to buccal crown height) and are splayed mesiodistally (Fig 6) resembling the condition in *Sivapithecus* (e.g., GSP 11786) and *Gorilla*, figured by Kupczik and Dean [39] but less like those of *Pan* (roots not as splayed or as long) or *Pongo* (roots not as splayed).

## Localities yielding *Sivapithecus*

*Sivapithecus* specimens are known from four regions in the Siwaliks: the Potwar Plateau of Pakistan, Ramnagar and Hari Talyangar in India [16], and the Churia Hills (one upper molar) in Nepal [40]. The Tapar specimen would constitute the first locality outside of the Siwaliks.

Most Potwar Plateau specimens come from three precisely dated intervals separated by temporal gaps of approximately 0.7 to 1.0 Ma [16, 25]:

- The Chinji Formation hominoid-bearing localities range between 12.7 and ~11.4 Ma. All Potwar specimens collected earlier in the twentieth century whose provenance is simply noted as “Chinji” probably fall within or close to this paleomagnetically-defined Chinji interval.
- The Y-GSP 311 locality lies within the upper Nagri Formation, dated to 10.1 Ma.
- The “U” Sandstone level [41], within the Dhok Pathan and Nagri Formations. These two formations are time-transgressive [25], so specimens from the upper levels of the Nagri Formation may be penecontemporaneous with specimens in the lowest parts of the Dhok Pathan Formation. The “U” Sandstone level is a prominent marker horizon dated to 9.3 to 9.2 Ma according to the GPTS revisions of Ogg et al. [42], with hominoid-bearing localities extending to 8.5 Ma [25, 43].

In the Siwalik group of India, most specimens are found at Hari Talyangar and a few from near Ramnagar. The Ramnagar area is broadly temporally equivalent to the Chinji Formation [16, 44]. Hari Talyangar’s primate-bearing level is penecontemporaneous with the “U” Sandstone level of the Potwar section centered at the base of Chron 4An of the GPTS, giving an age of ∼9.0 Ma [6].

It has been argued that *Sivapithecus* species may, in part, be time successive [9, 15, 16, 45]. Therefore, for the purposes of the analysis below, we follow Kelley [16] in clustering the *Sivapithecus* fossils into three above-mentioned temporal intervals: 1) a “Chinji group” (including the older Potwar plateau levels of Pakistan and Ramnagar localities; 2) the upper Nagri Formation locality Y-GSP 311 level from the Potwar Plateau; 3) the “U” Sandstone level from Pakistan with its roughly age-equivalent cluster of localities near Hari Talyangar, India.

## Species attribution

**Fig 7:**
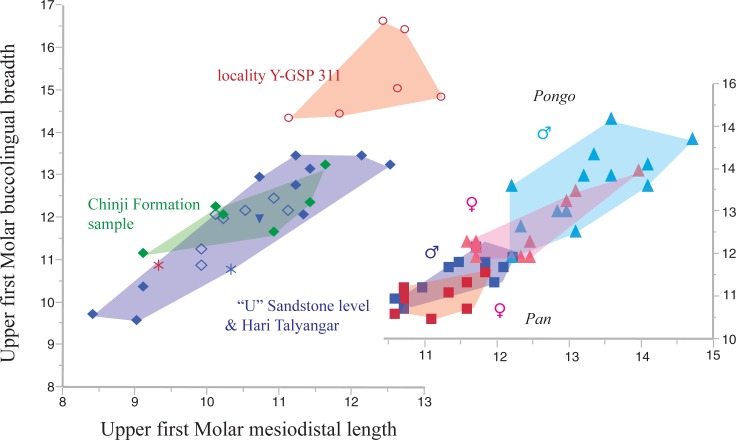
Bivariate plot of upper first molar mesiodistal length versus buccolingual breadth (in mm) for sample of *Sivapithecus* spp. from the Siwalik Series. Chinji Formation (green filled diamonds), Nagri/Dhok Pathan Formations (blue filled diamonds), Hari Talyangar specimens (open diamonds). Specimens from locality Y-GSP 311 (red circles) are *Sivapithecus parvada* Kelley, 1988. Inverted triangle is GSI D1, the type specimen of *Sivapithecus sivalensis* (Lydekker), 1879. Red asterisk is WIHG WIF/A 1099. Blue star is GSI D185 from Hari Talyangar, a part of the originally proposed hypodigm of *S*. *simonsi* [14]. Inset: dimensions of the upper first molars in a sample of *Pongo pygmaeus* (10 males and 16 females) and *Pan troglodytes* (13 males and 19 females of each; blue (male) and red (female) triangles and blue and red squares, respectively).

**Fig 8:**
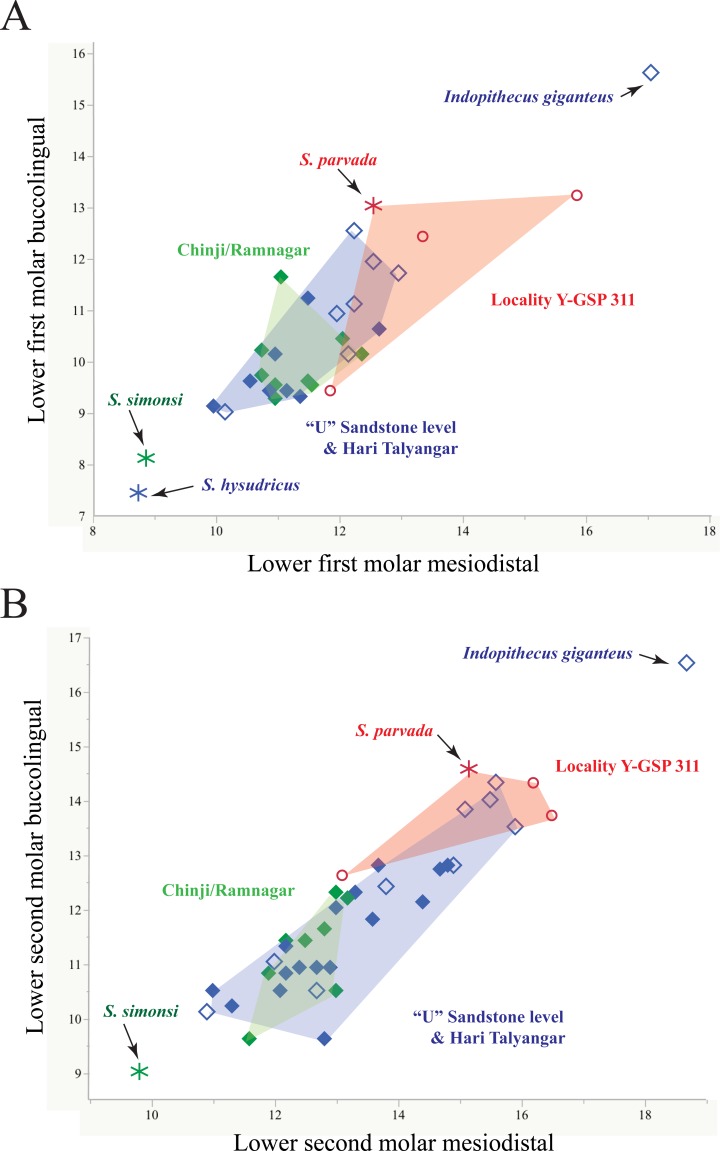
Bivariate plots of lower cheek teeth of *Sivapithecus* spp. A. Lower first lower molar mesiodistal length versus buccolingual breadth (in mm). B. Lower second lower molar mesiodistal length versus buccolingual breadth (in mm). Symbols: Chinji Formation and Ramnagar (green filled diamonds), Nagri/Dhok Pathan Formations (blue filled diamonds) and Hari Talyangar localities (blue open diamonds), and locality Y-GSP 311 (red open circles). Asterisks denote type specimens of *Sivapithecus simonsi* (in green), *S*. *hysudricus* (in blue), and *S*. *parvada* (in red).

All *Sivapithecus* species have low, bunodont and thickly enameled molars. The upper and lower cheek teeth lack cingula. Formerly it was thought that there were morphological distinctions in the shape of the canines between *S*. *indicus* and *S*. *sivalensis* but now it is known that this distinction does not exist [46]. Currently, only overall size and tooth proportions, mandibular structure, and stratigraphic level are used to distinguish the taxa. In Fig 7 we compare the upper first molar sample from Chinji and Ramnagar with that from the younger “U” Sandstone level and Hari Talyangar. Our findings for metrics of the lower cheek teeth yield similar results (Fig 8), except as noted. As models to compare these size-shape plots, we include a similar plot for upper first molars of samples of extant *Pongo pygmaeus* and *Pan troglodytes*.

Before discussing the possible allocation of WIHG WIF/A 1099 to a species of *Sivapithecus*, the following summary is offered. For more background, one may consult Kay [14], Kelley & Pilbeam [47], and Kelley [16, 45]. With the possible exception of *Indopithecus giganteus* von Koenigswald 1950, there is general agreement that the large-bodied apes from the Siwalik Series of India and Pakistan belong to a single genus, *Sivapithecus*.

### Species names for *Sivapithecus*

We consider two possible species-naming strategies for the Indian and Pakistan *Sivapithecus* material. The first is a time-successive, or ‘stratophenetic’ species concept [48], which would recognize taxa of similar morphology at different stratigraphic levels; morphology is considered paramount but specimens of similar morphology that differ in stratigraphic horizon may be assigned to different species. The second alternative allows for the possibility that the same species can occur at various stratigraphic levels and geographic localities [49].

#### Names available for time-successive species

As noted above, most recent researchers recognize three time slices for *Sivapithecus* [9, 15, 16, 45]. If we accept the concept of separately-named time successive species the following names would apply:

- The geologically oldest species would be *S*. *indicus* (Pilgrim, 1910) (type specimen, GSI D 175, an isolated lower second molar), from the Chinji Formation.
- An intermediate-aged species would be *S*. *parvada* Kelley, 1988 (type specimen, BSPhG 1939 X4, a right and left mandibular corpus with left p3-4 and m2-3 and right c, p3 and m2). The type and all currently referred material come from Y-GSP 311 in the Nagri Formation [45].
- The youngest species would be *S*. *sivalensis* (type specimen, GSI D1, a maxilla with C-M3). GSI D1 reportedly came from near the village of Jabi [4]. While it is unclear precisely where Jabi is located because there are several villages of that name, the material associated with, or in the same collections as, the type specimen suggests an occurrence in the Dhok Pathan Formation. If so, this would certainly be a specimen of young age, perhaps roughly equivalent to the *Sivapithecus* material from the “U” Sandstone level (J. Barry, personal communication).

The possibility has been raised repeatedly that there is also a smaller species from Chinji, “U” Sandstone and Hari Talyangar levels, though not from the Y-GSP 311 locality. From the Chinji levels, the names *Sivapithecus punjabicus* (Pilgrim, 1910) (type specimen, GSI D 118/119, right and left mandibular fragments with m2 and m3 from Kundal Nala), and *Sivapithecus simonsi* Kay, 1981(type specimen, GSI D-298; field no. 618, from uppermost Chinji Horizon, Kundal Nala, Chinji [50], a mandible with p3-m2) are available.

From Potwar, “U” Sandstone level specimens and those from Hari Talyangar, a number of names could be applied to the smaller species. Pickford [13] suggests that *Sivapithecus* = ‘*Hylopithecus’ hysudricus* (Pilgrim, 1927) has priority. The type of *S*. *hysudricus* is a lower molar, GSI D 200, considered by Pickford to be an m1 (Pilgrim considered it to be an m2), from Hari Talyangar. Pickford’s comments correct several prior misreadings of Pilgrim’s paper. Simons and Pilbeam [51] claimed that Pilgrim [52] identified the tooth as a lower third molar and they comment that the specimen was embedded in a mandibular fragment; rather, Pilgrim identified the tooth as an m2, and mentions a distal interproximal wear facet, ruling out the possibility that it is an m3. Pilgrim does not mention that the tooth was associated in a mandibular fragment. The next available name for a small species in the younger levels is *Sivapithecus brevirostris* (Lewis, 1934) named for a right maxilla and premaxilla with P3-M2, an alveolus for the canine, and an I2 root (Y.P.M. No. 13799).

#### Recognizing species at various stratigraphic levels (stratophenetic scheme)

The size range of maxillary and mandibular molars of *Sivapithecus* is illustrated in Figs 7 and 8 (data in S1 Table). Several observations can be made.

- *Sivapithecus* specimens from Chinji, nominally assigned to *S*. *indicus* would appear to be no more variable than expected for a single moderately to highly dimorphic ape like *Pongo*. Extending these observations to the lower teeth (Fig 8), the *S*. *indicus* sample (which has name priority) would include *Sivapithecus punjabicus* at the smaller end. *Sivapithecus simonsi* (from lower teeth, Fig 8) is a clear outlier in the Chinji sample and may be a valid taxon in a stratophenetic scheme. Specimens from Ramnagar available to us, although geographically isolated from the Potwar sample, show no distinctive metric signal.
- Variation in specimens from Harvard locality Y-GSP 311 is consistent with a single dimorphic and larger variant of *Sivapithecus*. Specimens from this locality have been assigned to *S*. *parvada* by Kelley [45], a name used exclusively for specimens from Y-GSP 311.
- Turning to specimens from the youngest time interval (“U” Sandstone level and Hari Talyangar), *Indopithecus giganteus* is generally recognized as a very large species likely generically distinct from *Sivapithecus*. The size range of the remaining sample of maxillary and mandibular molars from the youngest interval is extremely broad, as illustrated in Figs 7 and 8. These specimens have been examined by several workers, most recently by Kelley [16], Scott et al. [53], and Pickford [13, 54]. Several points can be made: 1) The variation in the Hari Talyangar sample exceeds that of any living catarrhine and even exceeds that of *Lufengpithecus*. Scott et al. [53] acknowledge the extraordinary amount of variation in the sample and that two-species may be present. Importantly, neither Scott et al. nor Kelley included the very small *S*. *hysudricus* specimen (GSI D 200) in their analyses, which would even further increase the size range of the Hari Talyangar specimens. We therefore consider it likely, as did Kelley and Pilbeam [47] that there are two species represented in the sample, particularly if GSI D 200 is included (which they did not). 2) Sample variation in the Potwar sample is encompassed by the penecontemporaneous Hari Talyangar sample; there is no rationale, other than geography, to recognize different species from these two regions. Taken together, the Hari Talyangar and younger Potwar Plateau material increases the evidence for the presence of a small *Sivapithecus* species [13, 54]. BMNH M 15423 from the Nagri Formation according to Pilbeam [55] has estimated m1 or m2 lengths nearly identical to GSI D 200 from Hari Talyangar (see S1 Table).

In a stratophenetic scheme, the larger of the two species from the youngest level would be called *Sivapithecus sivalensis* and the smaller *S*. *hysudricus*.

#### Morphology-based species crossing time and space

Under this scenario, the null hypothesis would be that *Sivapithecus indicus* and *S*. *sivalensis*, despite the fact that they span a lengthy (and discontinuous) time interval (8.5 to 12.7 Ma), represent a single species in stasis because they have similar mean size, size range, proportions, and morphology. This is a plausible scenario, as noted by Kelley and Pilbeam [47]—*S*. *indicus* is from the Chinji and Ramnagar and *S*. *sivalensis* is from the Potwar “U” Sandstone level and similar-aged Hari Talyangar material. The two ‘species’ could represent a single similarly-sized species spanning the early Late Miocene. *S*. *parvada* and *S*. *simonsi/S*. *hysudricus* seem to be distinct outliers and will be discussed independently.

No compelling differences in dental, mandibular, or cranial anatomy convincingly separate *S*. *indicus* from *S*. *sivalensis*. Leaving aside two very small specimens (discussed below), the younger dental material from Hari Talyangar and the “U” Sandstone level assignable to *S*. *sivalensis* completely encompasses the size range of Chinji and Ramnagar specimens that would be assigned to *S*. *indicus*. (The canine structure of *S*. *indicus* is no longer considered distinctive as was formerly thought [46].) Other proportional differences in molar size between the material from the two temporal intervals have been proposed, but these seem to us as likely to be the result of individual variation as indicative of species differences between the stratigraphic levels. For example, differences in the proportions of m2 relative to m3 have been mentioned [16]. However, m3 length follows a positively allometric trend relative to m2 length in both the Chinji/Ramnagar and Nagri/Hari Talyangar samples (i.e., smaller specimens have relatively smaller m3s). Some differences in the palate between two specimens from the Chinji Formation (GSP 16075) and the Nagri Formation (GSP 15000), in particular the structure of the anterior nasal spine and crest [56], have been forwarded as specific or even generic differences, but others have dismissed these differences as individual variation rather than species differences [16].

Indirect lines of evidence make it plausible that *S*. *indicus* and *S*. *sivalensis* could represent a single lineage. First, the survival of a species with minimal change is documented in living hominoids. The Sumatran and Bornean species (or subspecies) of *Pongo* (*P*. *abelii* and *P*. *pygmaeus*) have been separate lineages for ~4 million years [57] but show very similar dental and gnathic morphology [35, 58]. Therefore, dental and gnathic stasis has to be considered a real possibility for Siwalik *Sivapithecus* as has been demonstrated for *Pongo*, the species of which span an equivalent time range to *Sivapithecus*.

Nor is it unusual for stasis to occur over long time spans in other Indo-Pakistan Miocene species. Flynn et al, [34] records 42 of 122 recorded mammalian species from the Siwaliks (34%) that survived 3 or more million years; 14 of 19 Siwalik families have a species that survive for 3.0 Myr or greater. Badgley et al.’s data [59] show the same thing: 37% of Siwalik taxa exceed the known temporal duration of *Sivapithecus* (based on recorded, not inferred, first appearance and last occurrence).

In short, given the failure to show convincing species-level differences in the morphology of *S*. *indicus* and *S*. *sivalensis*, the demonstrated longevity of living Asian great apes, and the common occurrence of long-lived mammalian species in the Miocene Siwaliks, there is no obvious reason why some portion of these younger specimens should not represent the same taxon as the Chinji and Ramnagar material. The type specimen of *S*. *sivalensis* (GSI D1) is probably from the Dhok Pathan Formation. In M1 size (and in molar proportions; see S1 Table for measurements), GSI D1 falls into the middle of the sample from the Chinji Formation and would have naming priority as a morphospecies. Thus, at the moment, we would entertain the hypothesis that *S*. *indicus* and *S*. *sivalensis* are the same species, with *S*. *sivalensis* (Lydekker 1879) having naming priority.

We have noted the occurrence of very small specimens from Chinji/Ramnagar and also from “U” Sandstone levels and Hari Talyangar. On available evidence, the two appear indistinguishable and represent the same small species. In this case, we would follow Pickford and colleagues and assign the name of the Hari Talyangar-based *Sivapithecus hysudricus* (Pilgrim, 1927) in priority over the Chinji level *S*. *simonsi* Kay, 1982.

#### Time successive species or stasis?

The two species pairs mentioned above—*Sivapithecus indicus* and *S*. *sivalensis* on one hand and *S*. *simonsi* and *S*. *hysudricus* on the other hand, are as yet virtually indistinguishable clusters of specimens exhibiting only slight morphological differences not exceeding the ranges expected among generally recognized single extant catarrhine species. That makes them good candidates for being discrete species lineages. The most salient diagnostic difference is geological age. Whether one wishes to recognize discrete time-successive lineage segments with different species names or apply single names to each proposed lineage as a whole is a matter of taste. We opt for the simpler scheme of recognizing the two clusters by single names—*Sivapithecus hysudricus* (Pilgrim, 1927) for the smaller species and *Sivapithecus sivalensis* (Pilgrim, 1910) for the larger. For the moment, we leave *S*. *parvada* Kelley, 1988 as a valid taxon found only at one stratigraphic level. A fourth species of Indo-Pakistan ape, *Indopithecus giganteus* from the Hari Talyangar area is also recognized.

Having reached these conclusions, it is important to emphasize the point mentioned by many who have studied Siwalik apes—allocation of individual specimens to one of the two species—*S*. *sivalensis* or *S*. *hysudricus* is difficult, as their size-ranges almost certainly overlap and the nondental material is as yet poorly known and non-diagnostic. This should not be surprising, as a similar phenomenon has been reported repeatedly in living Old World monkeys, where in some cases as many as three valid species of *Cercopithecus* form polyspecific groups easily recognized by coat color and pattern but largely overlapping in dental and gnathic morphology [60]. This is a practical difficulty for paleontologists but represents a biological reality [61].

### Identity of WIHG WIF/A 1099

The Kutch specimen is apparently penecontemporaneous with *Sivapithecus* from Hari Talyangar and the “U” Sandstone levels, which occur after the Asian appearance of *Hipparion* at 10.8 Ma [3]. The molars of WIHG WIF/A 1099 fall at the lower end of the size range for Hari Talyangar and “U” Sandstone specimens (Figs 7 and 8). The upper cheek tooth dimensions of WIHG WIF/A 1099 overlap the range such that the specimen could be allocated to either a small *S*. *sivalensis* or, by inference from the size of the uppers, a large *S*. *hysudricus*.

## Summary and conclusions

WIHG WIF/A 1099, collected in 2011 from basal Late Miocene deposits in the Kutch district in the state of Gujarat, western India, represents a southern range extension of the Miocene hominoid *Sivapithecus*. Associated specimens of *Hipparion* (*sensu lato*) indicate that it is post-*Hipparion* datum in age, younger that 10.8 Ma, a similar time range as the “U” Sandstone and Hari Talyangar levels of the Siwaliks. A review is offered of the complex taxonomy of Miocene Siwalik apes on the basis of which we recognize two apparent lineages that span the range of known *Sivapithecus*: a small species *S*. *hysudricus* and a medium sized species *S*. *sivalensis*. *S*. *parvada* and *Indopithecus giganteus* are two other valid Siwalik taxa.

WIHG WIF/A 1099 is the only known ape specimen from Gujarat and preserves only worn and eroded maxillary teeth embedded in a poorly preserved maxilla. From what can be discerned, this specimen resembles *Sivapithecus* in having thickly enameled cheek teeth that lack lingual cingula. In size, it falls toward the lower end of the size range for Siwalik *Sivapithecus*. WIHG WIF/A 1099 is best recognized as a large individual of *Sivapithecus hysudricus* or a small individual of *S*. *sivalensis*.

## Supporting information

S1 Table Dental dimensions of the maxillary and mandibular cheek teeth of *Sivapithecus* and extant ape specimens. *Sivapithecus* measurements by RFK; those of extant *Pongo* spp., and *Pan troglodytes* from Plavcan [35].
(XLSX)
