# Correction: First record of the Miocene hominoid *Sivapithecus* from Kutch, Gujarat state, western India

**DOI:** 10.1371/journal.pone.0217960

**Published:** 2019-05-31

**Authors:** Ansuya Bhandari, Richard F. Kay, Blythe A. Williams, Brahma Nand Tiwari, Sunil Bajpai, Tobin Hieronymus

There are errors in the captions for Fig 4, “WIHG WIF/A 1099, right maxilla preserving Canine-M2,” Fig 5, “Two high resolution micro-CT parasagittal sections of WIHG WIF/A 1099, with teeth identified,” and Fig 6, “Buccal view of WIHG WIF/A 1099, composite CT image.” Please see the complete, correct figure captions here.

**Fig 4:**
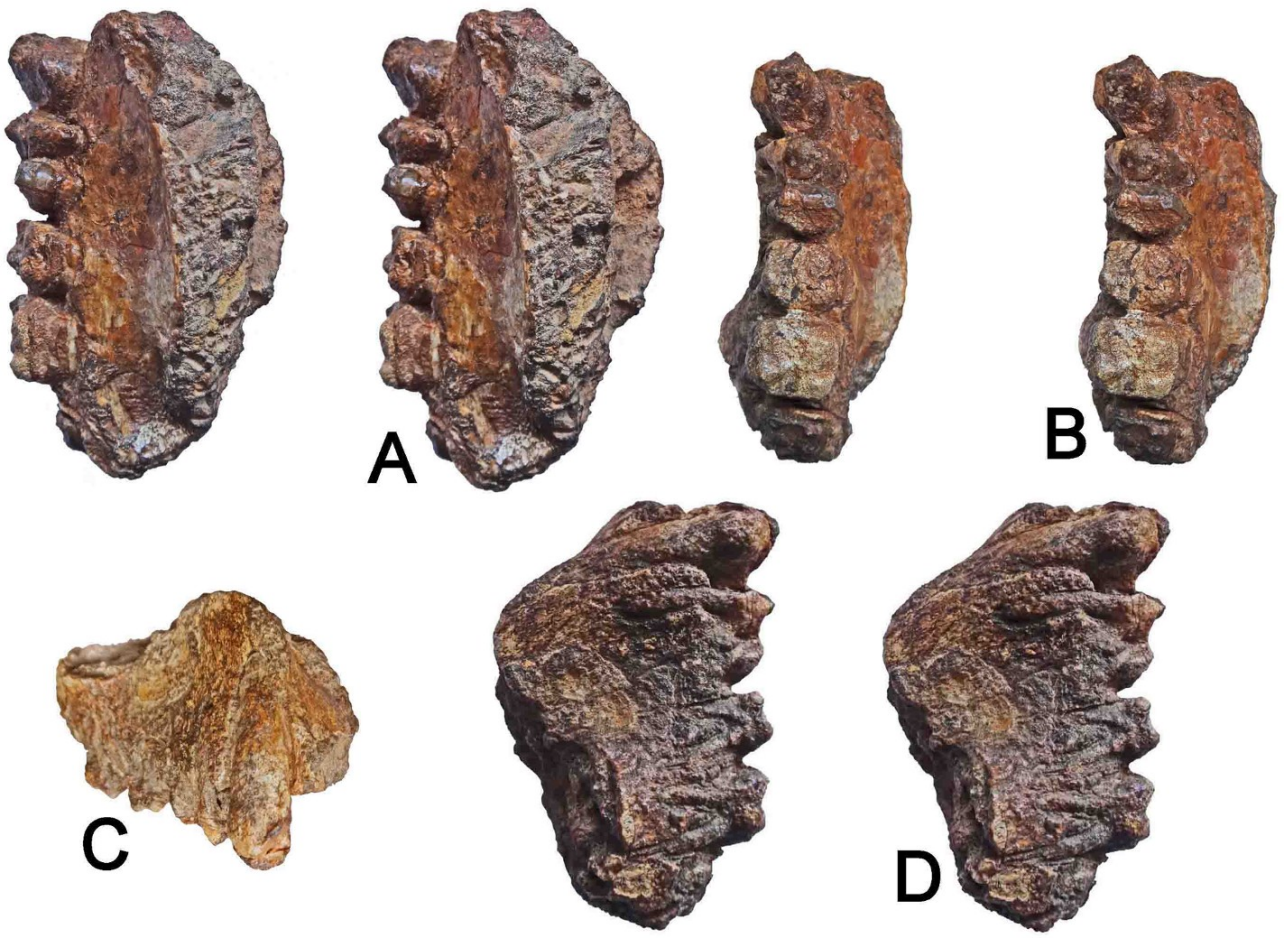
WIHG WIF/A 1099, right maxilla preserving Canine-M2. A. Stereopair of the palate and teeth viewed from lingual perspective. B. Stereopair of the palate and teeth viewed in occlusal perspective. C. The teeth and maxilla viewed frontally. D. Stereopair palate and teeth viewed from lateral (buccal) perspective. Image magnification is variable. Length of toothrow (C-M2) = 45.0 mm; specimen length = 57.5 mm. Photos courtesy of Martin Pickford.

**Fig 5:**
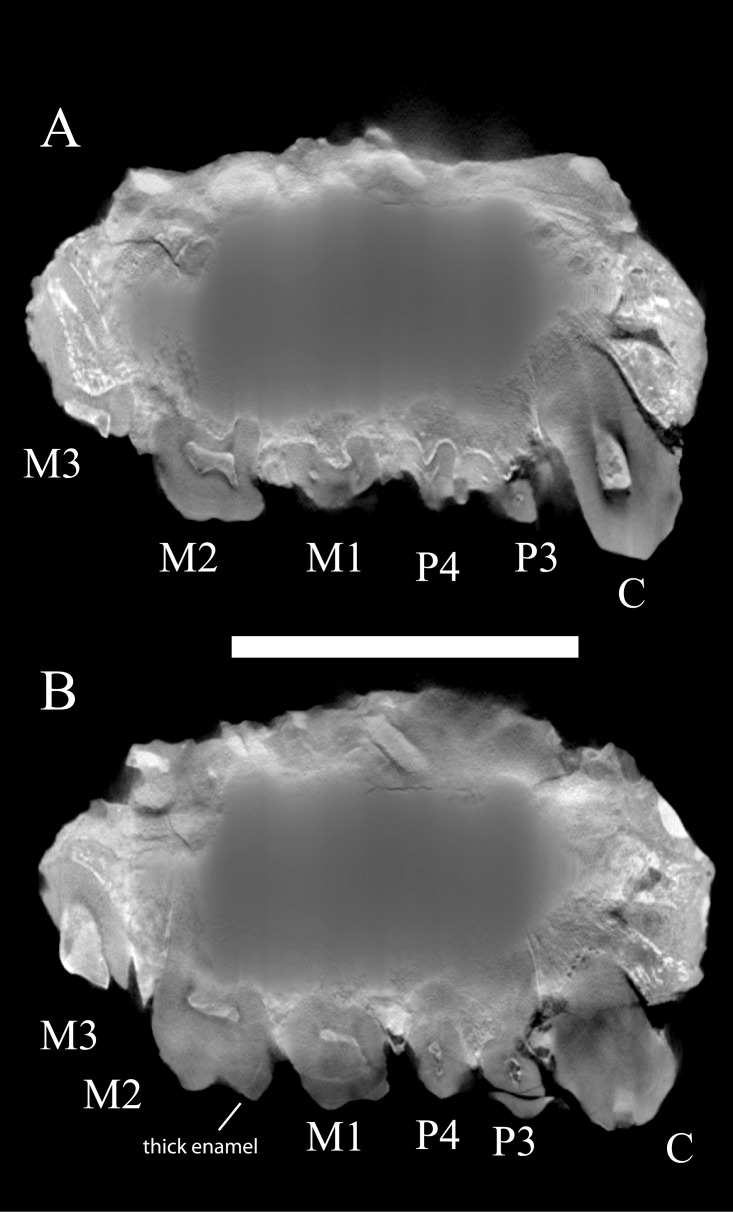
Two high resolution micro-CT parasagittal sections of WIHG WIF/A 1099, with teeth identified. A. The root structure of the canine. B. The enamel thickness on M1. Scale bar equals 15 mm.

**Fig 6:**
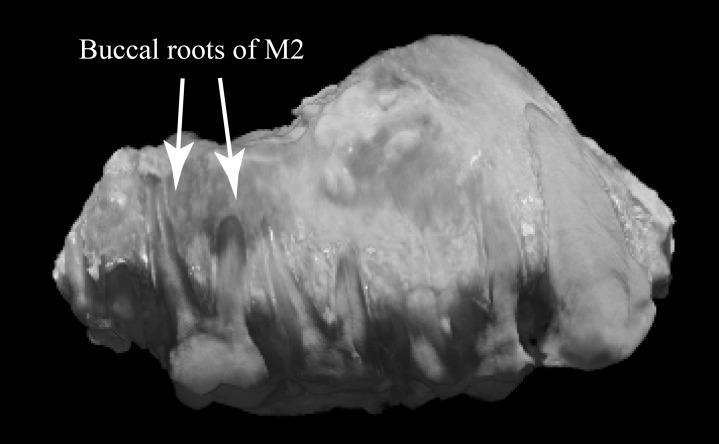
Buccal view of WIHG WIF/A 1099, composite CT image. Length of toothrow (C-M2) = 45.0 mm.
